# Real-World Outcomes of a Novel Botulinum Toxin A for Upper Face Aesthetics: Insights From Routine Clinical Practice

**DOI:** 10.1093/asjof/ojaf136

**Published:** 2025-10-24

**Authors:** Konstantin Frank, Nikolaus Duschek, Valentina Prinz, Michelle Friedrich, Mia Cajkovsky

## Abstract

**Background:**

Aging signs of the upper face result from complex interactions among facial muscles and include forehead lines, glabellar lines, and crow's feet. Botulinum neurotoxin type A (BoNT-A) injections are widely utilized to address these concerns. An optimal treatment strategy should consider the interplay between elevator and depressor muscles. Comprehensive treatment approaches targeting the comprehensive upper face are gaining popularity but remain underreported in the context of newer products such as letibotulinumtoxinA.

**Objectives:**

The aim of this study is to evaluate the efficacy and safety of a comprehensive upper-face treatment using letibotulinumtoxinA (Letybo) and to assess its effect on glabellar lines, forehead lines, and crow's feet using validated clinical scales.

**Methods:**

In this prospective, observational study, 20 participants (mean age: 35.8 ± 9.3 years) received standardized injections totaling 64 units. Treatment included 16 intramuscular injections: glabella (5 sites), forehead (5 sites), and crow's feet (3 per side), with 4 units (0.1 mL) per site. Efficacy was evaluated at baseline, Week 2, and Week 16 using the Facial Wrinkle Scale, Clinician's Dynamic Forehead Line Assessment Scale, and Clinician's Dynamic Crow's Feet Assessment Scale.

**Results:**

At Week 2, ≥2-point improvement was observed in 95% of glabellar, 90% of forehead, and 90% of crow's feet cases. At Week 16, ≥1-point improvements persisted in 70% to 80% of participants. Five participants (25%) required a touch-up at Week 2. No adverse drug reactions were reported.

**Conclusions:**

Comprehensive upper-face treatment with letibotulinumtoxinA showed meaningful, sustained aesthetic improvement with good tolerability. These findings support further research in larger populations.

**Level of Evidence: 4 (Therapeutic):**

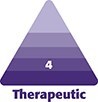

Botulinum neurotoxin A (BoNT/A) injections are among the most widely performed aesthetic procedures worldwide, with continuous advancements since their initial use for glabellar lines.^1-3^ The dynamic interplay between upper-face muscles necessitates a full treatment approach rather than isolated interventions to achieve more balanced, natural, and long-lasting aesthetic outcomes. The corrugator and procerus muscles contribute to vertical glabellar lines, whereas the orbicularis oculi muscle influences lateral canthal lines, and the frontalis muscle governs forehead lines.^4-6^ Treating only a single region can lead to asymmetries or suboptimal results over time and may also cause disproportionate aging effects or draw attention to untreated regions, diminishing overall facial harmony.

Although letibotulinumtoxinA has demonstrated efficacy in reducing glabellar line severity and is approved for the temporary improvement of moderate to severe vertical lines between the eyebrows, clinical practice often extends beyond this isolated use.^7^ A growing number of patients seek comprehensive upper-face rejuvenation, and physicians frequently adopt a comprehensive approach while simultaneously addressing forehead lines, glabellar lines, and crow's feet. Previous studies on other botulinum toxin formulations have confirmed both safety and efficacy in treating the entire upper face, leading to regulatory approvals for such applications.^8^ In their studies, the authors were able to showcase high patient satisfaction and the extended duration of efficacy, thereby highlighting the value of integrated upper-face treatment strategies. However, there is limited data on the real-world performance of letibotulinumtoxinA for comprehensive upper-face treatment.

In this prospective, noninterventional, single-center study, the authors aimed to assess the efficacy and safety of comprehensive upper-face treatment using letibotulinumtoxinA. By evaluating real-world outcomes in everyday clinical settings, the authors seek to contribute to the growing body of evidence supporting comprehensive upper-face treatment approaches, providing valuable insights into the aesthetic and functional benefits of this regimen.

## METHODS

### Study Sample

This was a noninterventional, observational study, meaning that no treatment protocols, randomization, or additional procedures were imposed beyond those of standard clinical care. All treatments were performed at the discretion of the physician as part of routine practice. This study was conducted to assess the efficacy and safety of comprehensive upper-face treatment using letibotulinumtoxinA, conducted between January 29, 2024, and September 9, 2024. The study population consisted of patients who sought routine botulinum toxin treatment at the clinic and fulfilled the general eligibility requirements for treatment. Participants were enrolled as part of standard clinical practice, and informed consent was obtained before treatment. All patients were treated in accordance with regional laws, good clinical practice, and the Declaration of Helsinki.^9^ Participants were eligible for inclusion in the study if they met all of the following criteria: (1) aged 18 years or older at the time of treatment; (2) presented with at least mild glabellar lines at maximum frown, mild dynamic forehead lines at maximum eyebrow elevation, or mild crow's feet at maximum smile; (3) had a stable medical condition with no uncontrolled systemic disease; and (4) provided signed informed consent and agreed to routine follow-up visits at 2 weeks and 4 months. Exclusion criteria included: (1) pregnancy or breastfeeding; (2) diagnosis of a neuromuscular disorder such as myasthenia gravis or Eaton–Lambert syndrome; (3) known impairment of blood coagulation; (4) the presence of eyelid ptosis; (5) known allergy to botulinum toxin or human albumin (blood protein); (6) active infection or inflammation at the intended injection sites; and (7) treatment with botulinum toxin in the upper face within the 16 weeks preceding study enrollment. The study received a positive vote from the Ethics Committee of Vienna under the reference number EK 23-077-0523.

### Treatment Approach

The treatment approach targeted the upper face, including the glabellar region, forehead, and lateral canthal lines. The dosing and injection sites were based on the standardized clinical routine practice, respecting individual anatomical considerations, ensuring a balanced modulation of facial muscles to achieve optimal aesthetic outcomes while preserving natural expressions.

### Injection Procedure

**Figure 1.**
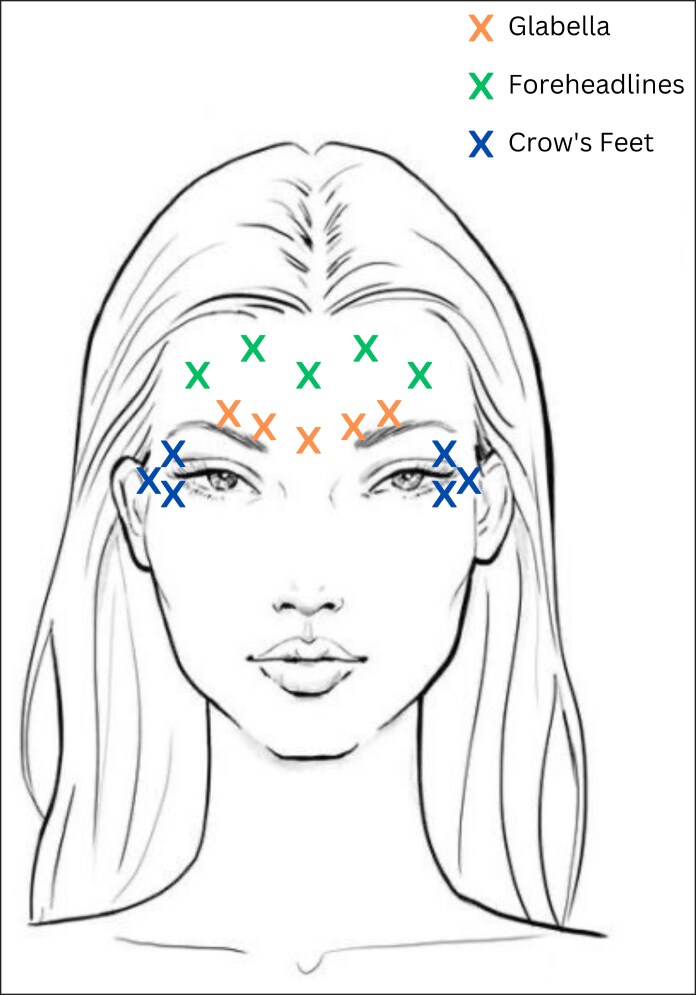
Injection scheme for the comprehensive upper face treatment.

All injections were administered by trained and experienced physicians using letibotulinumtoxinA (Letybo Croma Pharma GmbH, Leobendorf, Austria). LetibotulinumtoxinA (50 U vial) was reconstituted with 1.25 mL of sterile 0.9% sodium chloride (saline) to achieve a final concentration of 4 units per 0.1 mL. Injections were administered using a 1 mL syringe fitted with a 30G needle. A standardized intramuscular injection pattern was followed: 2 injections per corrugator supercilii muscle, 1 injection in the procerus muscle, 5 injections in the frontalis muscle, and 3 injections per side in the lateral orbicularis oculi muscle for crow's feet. Each site received 0.1 mL (4 U) of product (Figure 1). The total dose administered per patient was 64 units across 16 injection sites. If necessary, a touch-up treatment was performed at Week 2 at the physician's discretion to refine the results.

### Clinical Severity Grading

**Figure 2.**
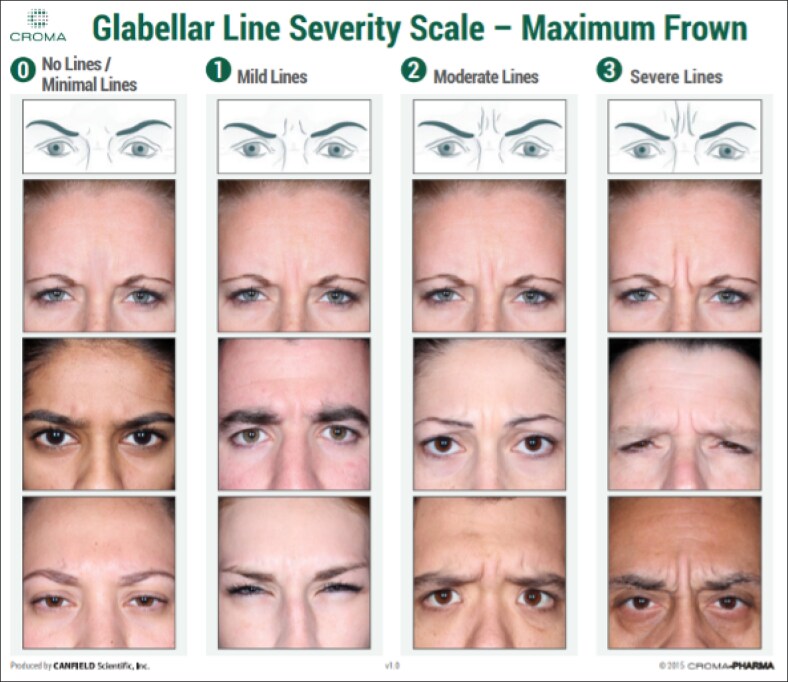
Facial Wrinkle Scale (FWS).

**Figure 3.**
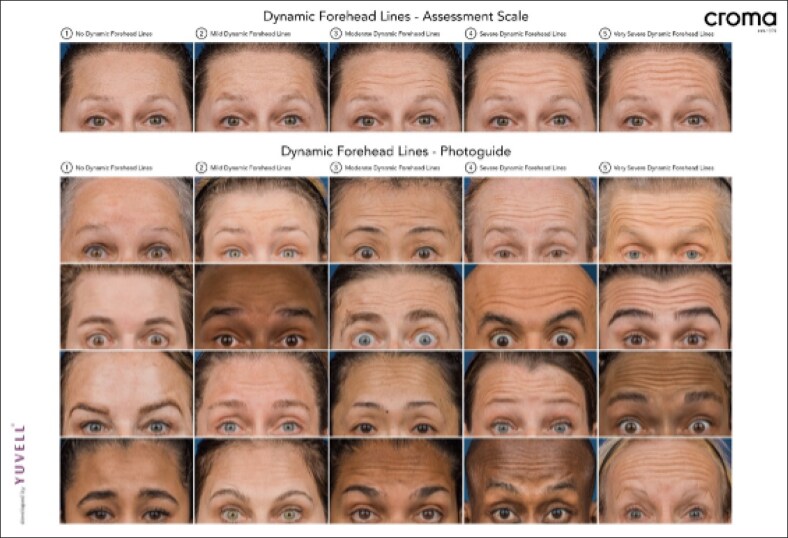
Croma Dynamic Forehead Line Assessment Scale (CDFLAS).

**Figure 4.**
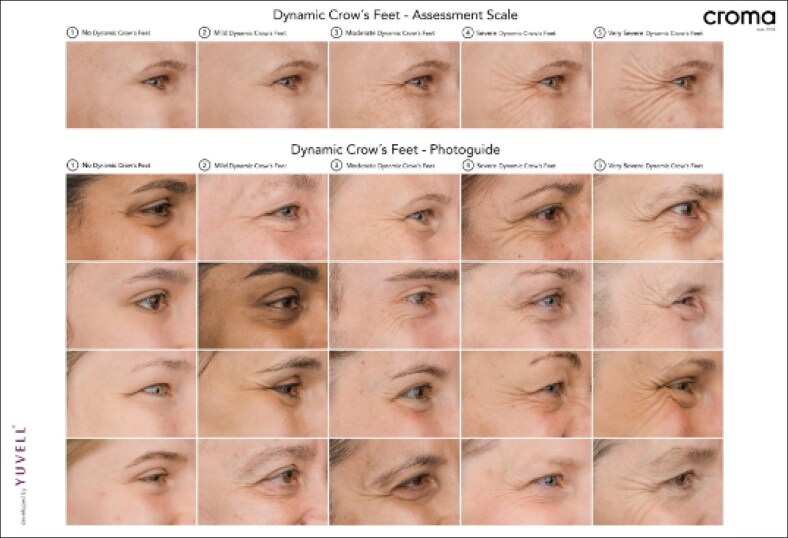
Croma Dynamic Crow's Feet Assessment Scale (CDCFAS).

The severity of upper-face lines was assessed dynamically using the Croma Dynamic Forehead Line Assessment Scale (CDFLAS) for forehead lines, the Facial Wrinkle Scale (FWS) for glabellar lines, and the Croma Dynamic Crow's Feet Assessment Scale (CDCFAS) for lateral canthal lines (Figures 2-4).^10-12^ These validated photonumeric scales—4-point (FWS) and 5-point (CDCFAS and CDFLAS)—are commonly utilized in clinical practice and help educate patients by visually communicating treatment goals and expected outcomes. All assessments were performed live by the principal investigator to ensure consistency and were conducted at baseline, the 2-week follow-up, and at Week 16. Standardized photographs were taken at each visit using the VECTRA M3 3D imaging system (Canfield Scientific Inc., Parsippany, NJ) under controlled lighting and positioning conditions to document facial expressions at maximum frown, eyebrow elevation, and full smile. At the 16-week follow-up, participants rated their treatment satisfaction using a numerical rating scale from 1 (very satisfied) to 5 (very unsatisified).

### Statistical Analysis

As a noncomparative, observational study without formal hypothesis testing, no statistical sample size calculation was performed. A total of 20 participants were enrolled based on feasibility and in line with common practice in small postmarket studies aiming to generate descriptive clinical data. Although the sample size is not sufficient to support inferential conclusions, it allows for preliminary assessment of treatment performance and tolerability in a real-world setting. Collected data were summarized using descriptive statistics. Safety analyses were conducted in the safety population, defined as all participants who received at least 1 injection of letibotulinumtoxinA. The intention-to-treat (ITT) population included all participants who received the investigational device and had at least 1 posttreatment assessment, whereas the per-protocol (PP) population included those who completed the investigation without major protocol deviations. Because no participants discontinued, the ITT and PP populations were identical. The primary efficacy endpoint was defined as the proportion of participants achieving a ≥1-point improvement from baseline at Week 2 in all 3 treatment areas (glabellar lines, forehead lines, and crow's feet). Secondary endpoints included the proportion of participants achieving a ≥2-point improvement, an FWS score of 0 or 1, and maintenance of improvements at Week 16, as assessed by the FWS, the CDFLAS, and the CDCFAS.

## RESULTS

### Overall Findings

The primary endpoint—defined as a ≥1 point improvement at Week 2 relative to baseline in all treated regions—was achieved by all 20 participants (100%). Secondary endpoints included the proportion of participants with an FWS score of 0 or 1 with a ≥2-point improvement and similar improvements in CDFLAS and CDCFAS at Weeks 2 and 16. At Week 2, a ≥2-point improvement was observed in 95% for glabellar lines, 90% for forehead lines, and 90% for crow's feet. By Week 16, a ≥1-point improvement was maintained in 80% for glabellar lines, 70% for forehead lines, and 80% for crow's feet, whereas a ≥2-point improvement was observed in 45%, 50%, and 20% of participants, respectively. Five participants (25%) required a touch-up at Week 2. Treatment satisfaction was high, with 90% of participants rating their experience with the highest score (1) on a 5-point scale. The mean follow-up time was 16 weeks with a standard deviation (SD) of 0.0 weeks (Table 1).

**Table 1.** Demographic Data of the Investigated Participants

	Safety analysis set	ITT	PPP
No. of participants	20	20	20
Age (years)			
*n*	20	20	20
Mean ± SD	35.8 ± 9.3	35.8 ± 9.3	35.8 ± 9.3
Median	36.0	36.0	36.0
Q1, Q3	28.0, 41.8	28.0, 41.8	28.0, 41.8
Min, max	22.0, 53.0	22.0, 53.0	22.0, 53.0
Gender			
*n*	20	20	20
Female	19 (95%)	19 (95%)	19 (95%)
Male	1 (5%)	1 (5%)	1 (5%)
BMI (kg/m^2^)			
*n*	20	20	20
Mean ± SD	22.1 ± 3.2	22.1 ± 3.2	22.1 ± 3.2
Median	20.9	20.9	20.9
Q1, Q3	19.6, 23.0	19.6, 23.0	19.6, 23.0
Min, max	18.6, 28.6	18.6, 28.6	18.6, 28.6
Height (cm)			
*n*	20	20	20
Mean ± SD	167.8 ± 8.0	167.8 ± 8.0	167.8 ± 8.0
Median	165.0	165.0	165.0
Q1, Q3	162.8, 172.8	162.8, 172.8	162.8, 172.8
Min, max	158.0, 189.0	158.0, 189.0	158.0, 189.0
Weight (kg)			
*n*	20	20	20
Mean ± SD	62.1 ± 9.6	62.1 ± 9.6	62.1 ± 9.6
Median	60.0	60.0	60.0
Q1, Q3	55.0, 67.0	55.0, 67.0	55.0, 67.0
Min, max	50.0, 84.0	50.0, 84.0	50.0, 84.0

PPP, per-protocol population; SD, standard deviation.

No adverse events (AEs) were reported throughout the study.

### Dynamic Glabellar Line Severity (FWS)

At baseline, the mean FWS score for glabellar lines was 2.4 ± 0.6. By Week 2, all participants had at least a 1-point improvement, with 95% achieving a ≥2-point improvement. The mean FWS score dropped to 0.0 ± 0.0 at Week 2, indicating complete resolution in all participants. The mean decrease compared with baseline was −2.4 ± 0.6 (95% CI, −2.7 to −2.2).

**Figure 5.**
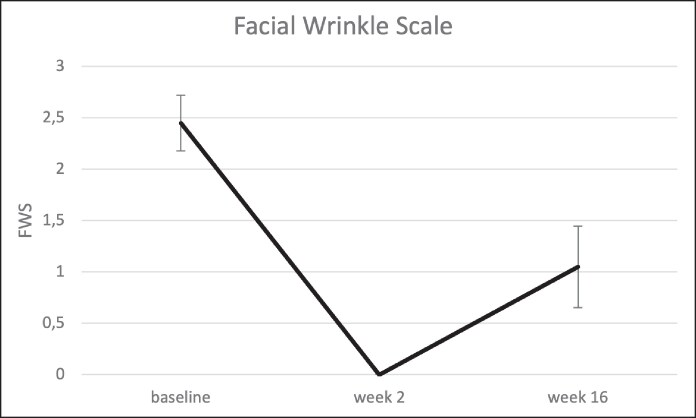
Facial Wrinkle Scale (FWS) at baseline, 2 weeks, and 16 weeks after letibotulinumtoxinA injection into the glabella. Bars indicate the standard error of means.

**Figure 6.**
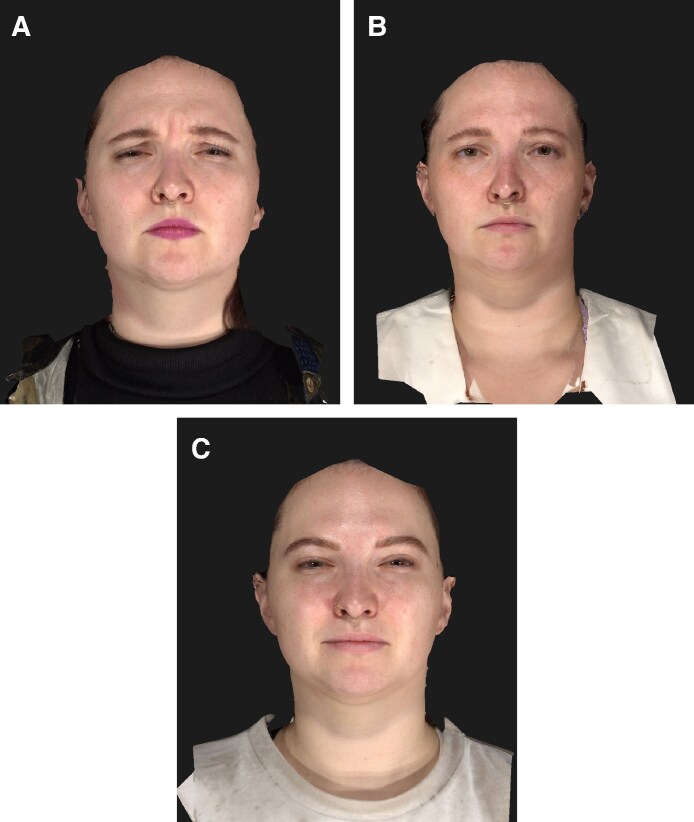
Clinical photographs of a 28-year-old female patient at (A) baseline, (B) 2 weeks, and (C) 16 weeks after letibotulinumtoxinA injection into the glabella. Maximal frown.

**Figure 7.**
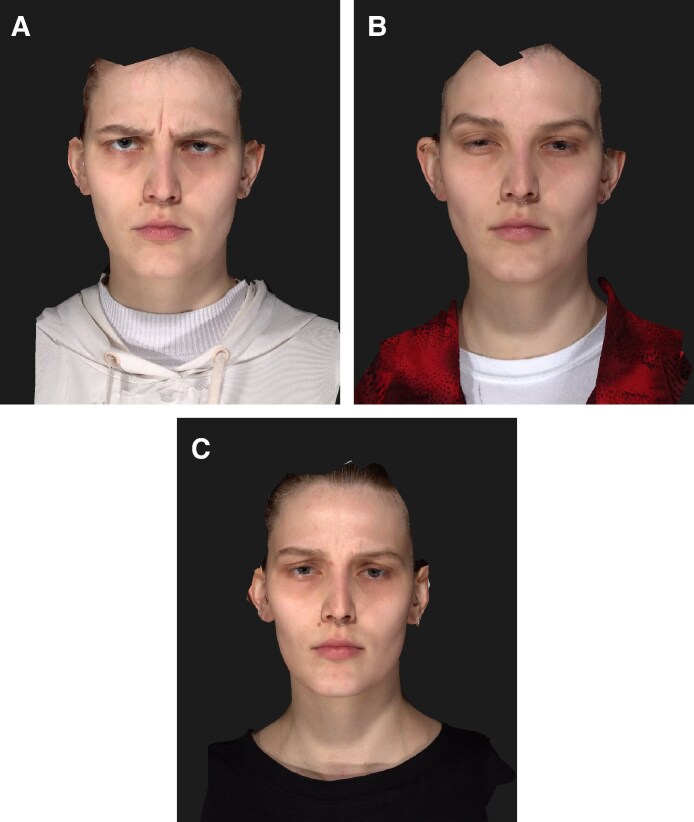
Clinical photographs of a 25-year-old female patient at (A) baseline, (B) 2 weeks, and (C) 16 weeks after letibotulinumtoxinA injection into the glabella. Maximal frown.

By Week 16, the mean FWS score was 1.0 ± 0.9, with 80% of participants maintaining at least a 1-point improvement, whereas 45% retained a ≥2-point improvement. The mean decrease compared with baseline was −1.4 ± 1.0 (95% CI, −1.9 to −0.9; Figures 5-7) (Table 2).

**Table 2.** Facial Wrinkle Scale (FWS)

	Baseline	Week 2	Week 16	Week 2 vs Baseline	Week 16 vs Baseline
*n*	20	20	20	20	20
Mean ± SD	2.4 ± 0.6	0.0 ± 0.0	1.0 ± 0.9	−2.4 ± 0.6	−1.4 ± 1.0
95% CI mean	2.2, 2.7	0.0, 0.0	0.6, 1.5	−2.7, −2.2	−1.9, −0.9
Median	2.5	0.0	1.0	−2.5	−1.0
Q1, Q3	2.0, 3.0	0.0, 0.0	0.0, 2.0	−3.0, −2.0	−2.0, −1.0
Min, max	1.0, 3.0	0.0, 0.0	0.0, 3.0	−3.0, −1.0	−3.0, 0.0

SD, standard deviation.

### Dynamic Forehead Line Severity (CDFLAS)

The CDFLAS score was 3.7 ± 0.7 (95% CI, 3.4-4.0] at baseline. By Week 2, all participants had a ≥1-point improvement, with 90% achieving a ≥2-point improvement, and the mean score reduced to 1.2 ± 0.5 (95% CI, 1.0-1.4). The mean decrease compared with baseline was −2.5 ± 0.6 (95% CI, −2.8 to −2.2).

**Figure 8.**
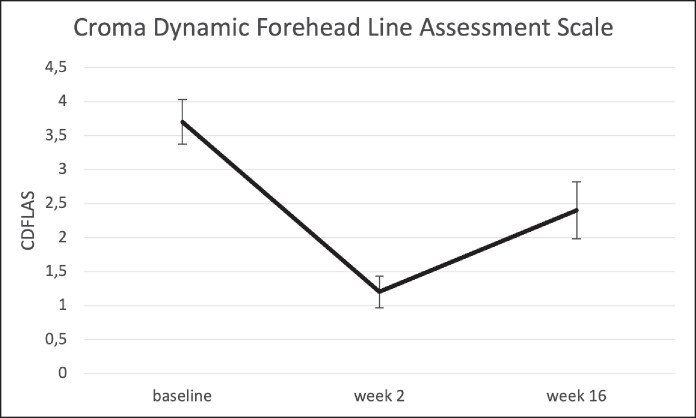
Croma Dynamic Forehead Line Assessment Scale (CDFLAS) at (A) baseline, (B) 2 weeks, and (C) 16 weeks after letibotulinumtoxinA injection into the forehead. Bars indicate the standard error of means.

**Figure 9.**
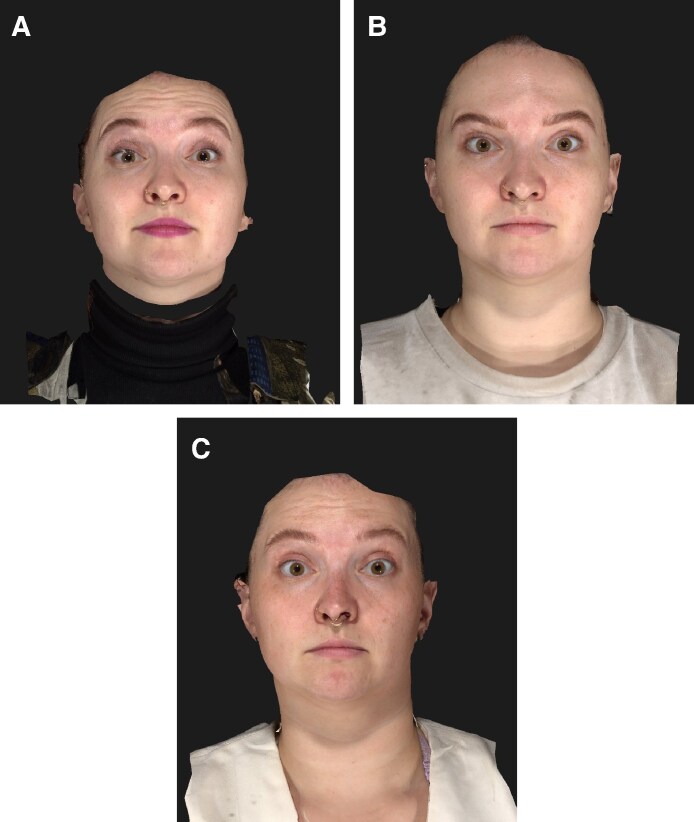
Clinical photographs of a 28-year-old female patient at (A) baseline, (B) 2 weeks, and (C) 16 weeks after letibotulinumtoxinA injection into the forehead. Maximal eyebrow elevation.

**Figure 10.**
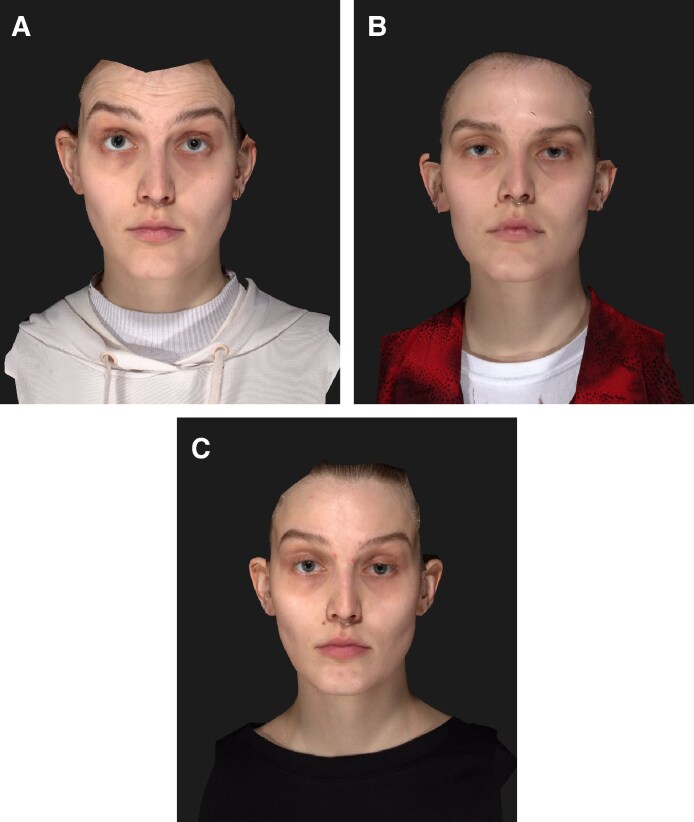
Clinical photographs of a 25-year-old female patient at (A) baseline, (B) 2 weeks, and (C) 16 weeks after letibotulinumtoxinA injection into the forehead. Maximal eyebrow elevation.

By Week 16, the mean CDFLAS score was 2.4 ± 0.9 ([95% CI, 2.0 to 2.8), with 70% of participants maintaining a ≥1-point improvement, whereas 50% retained a ≥2-point improvement. The mean decrease compared with baseline was −1.3 ± 1.3 (95% CI, −1.9 to −0.7; Figures 8-10) (Table 3).

**Table 3.** Croma Dynamic Forehead Line Assessment Scale (CDFLAS)

	Baseline	Week 2	Week 16	Week 2 vs Baseline	Week 16 vs Baseline
*n*	20	20	20	20	20
Mean ± SD	3.7 ± 0.7	1.2 ± 0.5	2.4 ± 0.9	−2.5 ± 0.6	−1.3 ± 1.3
95% CI mean	3.4, 4.0	1.0, 1.4	2.0, 2.8	−2.8, −2.2	−1.9, −0.7
Median	4.0	1.0	2.0	−3.0	−1.5
Q1, Q3	3.0, 4.0	1.0, 1.0	2.0, 3.0	−3.0, −2.0	−2.0, 0.0
Min, max	2.0, 5.0	1.0, 3.0	1.0, 4.0	−3.0, −1.0	−3.0, 2.0

SD, standard deviation.

### Dynamic Crow's Feet Severity (CDCFAS)

At baseline, the CDCFAS score was 3.8 ± 0.9 (95% CI, 3.4 to 4.3). By Week 2, all participants had at least a 1-point improvement, with 90% achieving a ≥2-point improvement, and the mean score reduced to 1.2 ± 0.4 (95% CI, 1.0 to 1.5). The mean decrease compared with baseline was −2.6 ± 0.9 (95% CI, −3.0 to −2.2).

**Figure 11.**
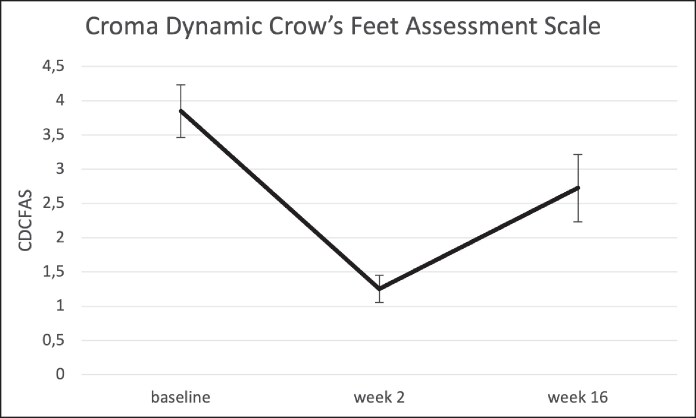
Croma Dynamic Crow's Feet Assessment Scale (CDCFAS) at baseline, 2 weeks and 16 weeks after letibotulinumtoxinA injection into the crow's feet region. Bars indicate the standard error of means.

By Week 16, the mean CDCFAS score was 2.7 ± 1.1 (95% CI, 2.2 to 3.2), with 80% of participants maintaining at least a 1-point improvement, whereas 20% retained a ≥2-point improvement. The mean decrease compared with baseline was −1.1 ± 0.8 (95% CI, −1.5 to −0.8; Figure 11, Supplemental Figures 1, 2) (Table 4).

**Table 4.** Croma Dynamic Crow's Feet Assessment Scale (CDCFAS)

	Baseline	Week 2	Week 16	Week 2 vs Baseline	Week 16 vs Baseline
*n*	20	20	20	20	20
Mean ± SD	3.8 ± 0.9	1.2 ± 0.4	2.7 ± 1.1	−2.6 ± 0.9	−1.1 ± 0.8
95% CI mean	3.4, 4.3	1.0, 1.5	2.2, 3.2	−3.0, −2.2	−1.5, −0.8
Median	4.0	1.0	2.8	−2.8	−1.0
Q1, Q3	3.5, 4.0	1.0, 1.2	2.0, 3.1	−3.0, −2.0	−1.5, −1.0
Min, max	2.0, 5.0	1.0, 2.0	1.0, 5.0	−4.0, −1.0	−3.0, 0.0

SD, standard deviation.

### Touch-Ups

Five participants (25%) required a touch-up at the 2-week visit. Touch-ups were most frequently administered in the forehead region. The mean ± SD doses per region were as follows: forehead: 11.0 ± 7.9 units; crow's feet (right): 2.8 ± 1.5 units; and crow's feet (left): 3.0 ± 1.0 units. The glabellar region required touch-up in only 1 case (5 units). Not all sites were retreated in each case; touch-ups were individualized based on residual muscle activity and aesthetic evaluation.

## DISCUSSION

Based on the results of this study, the authors highlight the importance of a combined multiregion approach to treat the upper third of the face with botulinum toxin A, demonstrating significant improvements in both wrinkle severity and skin movement dynamics. By targeting multiple facial regions—specifically the glabellar lines, forehead, and crow's feet—this approach addresses the coordinated muscle activity responsible for the formation of typical aging signs in the upper face. The glabellar region, in particular, is known for its muscle activity during frowning, whereas the forehead lines emerge from eyebrow elevation, and crow's feet result from smiling and squinting.^13,14^

The injection sites utilized in this study followed standard patterns commonly applied with BoNT-A products in the treatment of glabellar lines, forehead lines, and crow's feet. The approach was based not on novel injection points, but on the concept of treating the upper face as a functional and aesthetic unit, aiming for balanced outcomes and avoiding disharmony that may occur when only 1 region is addressed.^15^ Treating the entire upper face provides a more harmonious result that better reflects the natural movement and expression of the face, offering a more youthful and refreshed appearance.^16,17^

The combination approach, treating multiple regions simultaneously, leads to an improvement in the severity of wrinkles in different regions and is particularly effective as it minimizes the risk of over-treatment in a single region, ensuring that the treatment is tailored to the individual's unique facial structure and muscle dynamics (Table 4).

An important aspect of this study is its applicability across different ages, because the population included both younger and older participants. Furthermore, the authors found no significant differences between naive and non-naive patients, suggesting that previous experience with neuromodulator treatments does not influence the effectiveness or safety of the treatment investigated herein.^18^

No AEs were reported in this cohort of 20 participants, suggesting that letibotulinumtoxinA was well tolerated in this small real-world sample. The authors of this real-world study provide observational insights into the use of letibotulinumtoxinA for comprehensive treatment of the upper face. Although the injection strategy is based on standard clinical practice established with other BoNT-A products, its application to Letybo in this context remains off-label and should be interpreted with appropriate caution.

Although the authors of this study provide valuable insights into the efficacy and safety of a comprehensive upper-face treatment using letibotulinumtoxinA, several limitations should be acknowledged. This was a noninterventional study conducted at a single private clinic with a relatively small sample size of 20 participants. The small sample size (*n* = 20) and lack of a comparator or control group limit the generalizability of our findings. The observational design was not intended for hypothesis testing or regulatory conclusions but to explore real-world outcomes of comprehensive upper-face treatment using letibotulinumtoxinA. As such, results should be interpreted descriptively and considered hypothesis generating. Further large-scale, controlled studies are needed to validate these observations across diverse populations. In this study, the authors did not include a control or comparator group (eg, placebo or standard BoNT/A treatment in isolated regions). As a result, improvements in wrinkle severity and skin movement could not be directly compared with alternative approaches or to untreated controls, limiting the ability to attribute observed effects exclusively to the comprehensive upper-face treatment protocol. Although the authors of the study demonstrated sustained effects up to 16 weeks, longer follow-up is necessary to evaluate the full duration of action and potential delayed AEs. Additional limitations include the fact that all wrinkle severity assessments were performed by the treating physician, which may introduce observer bias, and that no formal statistical analysis was conducted because of the exploratory nature and limited sample size of the study. The treatment utilized in this study is based on clinical principles already validated in previous large-scale studies using established botulinum toxins. This investigation does not introduce a novel injection pattern but rather evaluates its use with letibotulinumtoxinA, a product for which real-world, multiregional data are limited. The findings support the reproducibility of comprehensive upper-face treatment strategies in routine practice when using a newer BoNT-A formulation.

## CONCLUSIONS

In this preliminary study, the authors demonstrated that comprehensive upper-face treatment with letibotulinumtoxinA resulted in measurable improvements in dynamic wrinkle severity across the glabellar lines, forehead lines, and crow's feet regions. These improvements were observed at 2 weeks and were largely maintained through 16 weeks. No AEs were reported, and overall treatment satisfaction was high among participants. Although the same standardized injection protocol was applied to all participants, the consistent responses across multiple anatomical regions suggest that simultaneous treatment may support aesthetic balance. These findings support the feasibility and tolerability of comprehensive upper-face treatment with letibotulinumtoxinA in clinical practice and warrant further investigation in larger, controlled studies.

## Supplemental Material

This article contains supplemental material located online at https://doi.org/10.1093/asjof/ojaf136.

## Supplementary Material

ojaf136_Supplementary_Data
