# Supplementary figures and images for: Real-World Outcomes of a Novel Botulinum Toxin A for Upper Face Aesthetics: Insights From Routine Clinical Practice
Source: Aesthet Surg J Open Forum. 2025 Oct 24;7:ojaf136. doi: 10.1093/asjof/ojaf136 (PMC12663534; doi:10.1093/asjof/ojaf136)

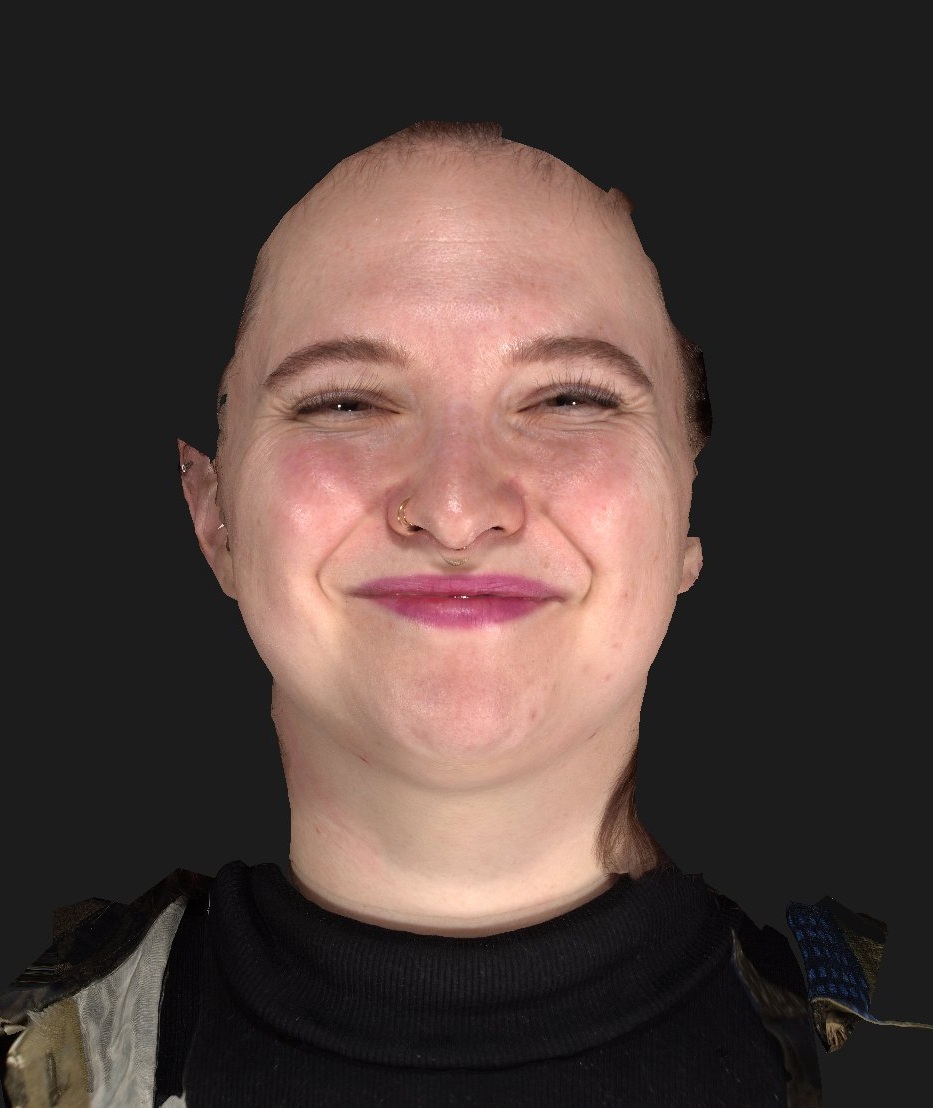

Supplement: ojaf136_Supplementary_Data [file ojaf136_supplementary_data.zip › Supplementary Figure_1A.jpg]

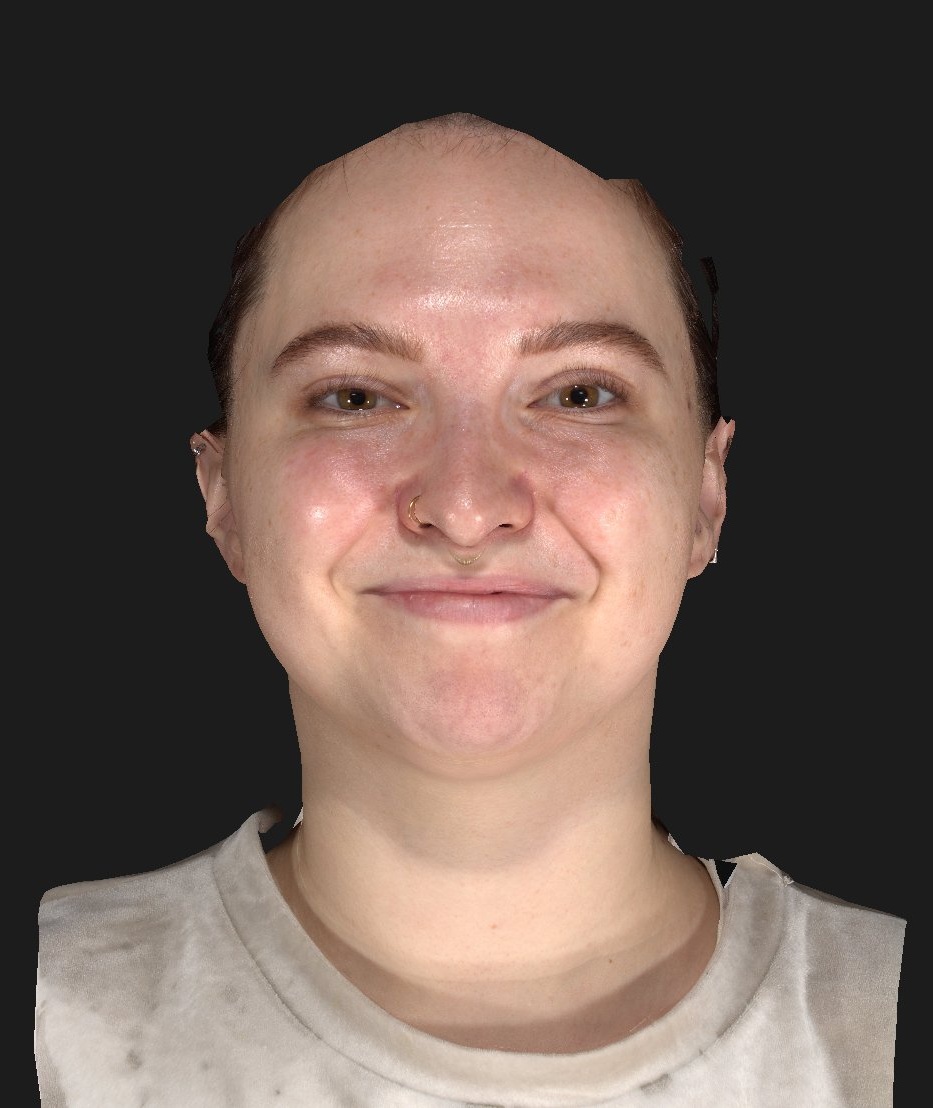

Supplement: ojaf136_Supplementary_Data [file ojaf136_supplementary_data.zip › Supplementary Figure_1B.jpg]

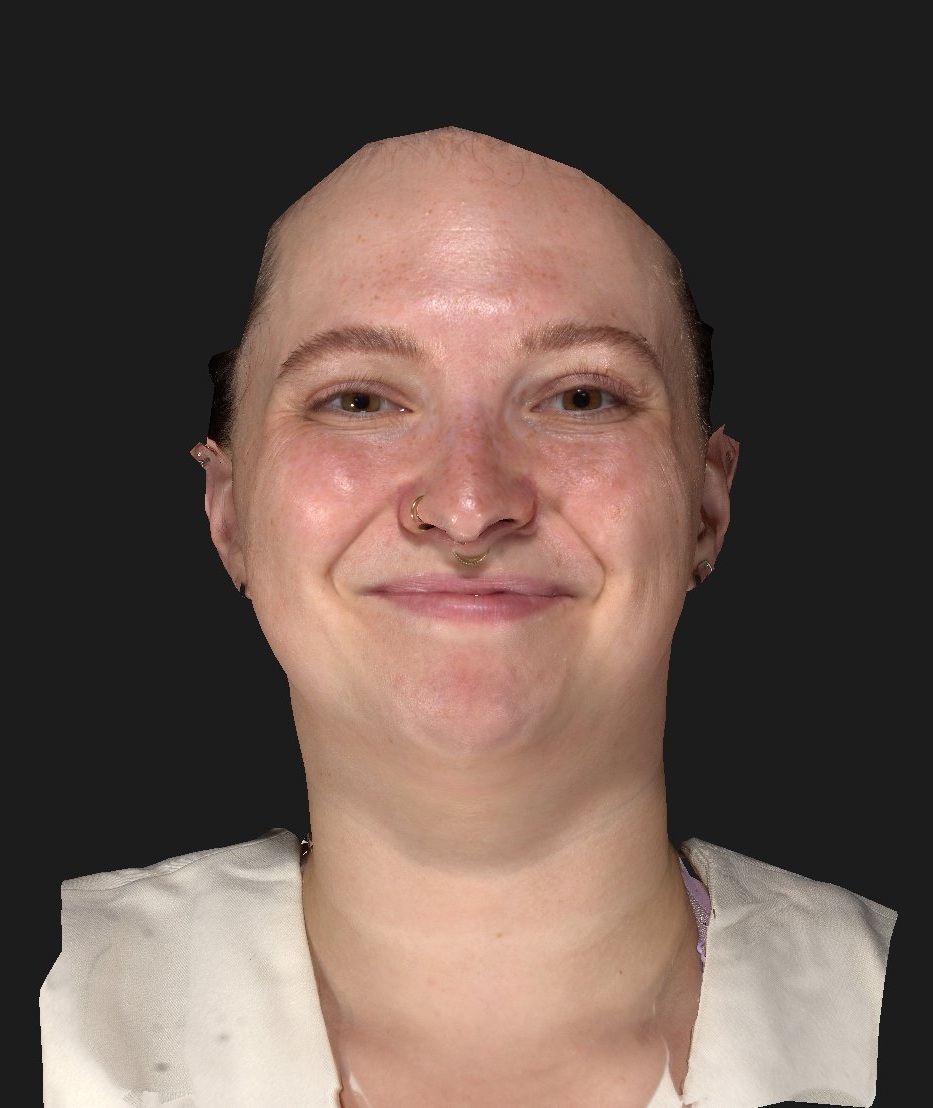

Supplement: ojaf136_Supplementary_Data [file ojaf136_supplementary_data.zip › Supplementary Figure_1C.jpg]

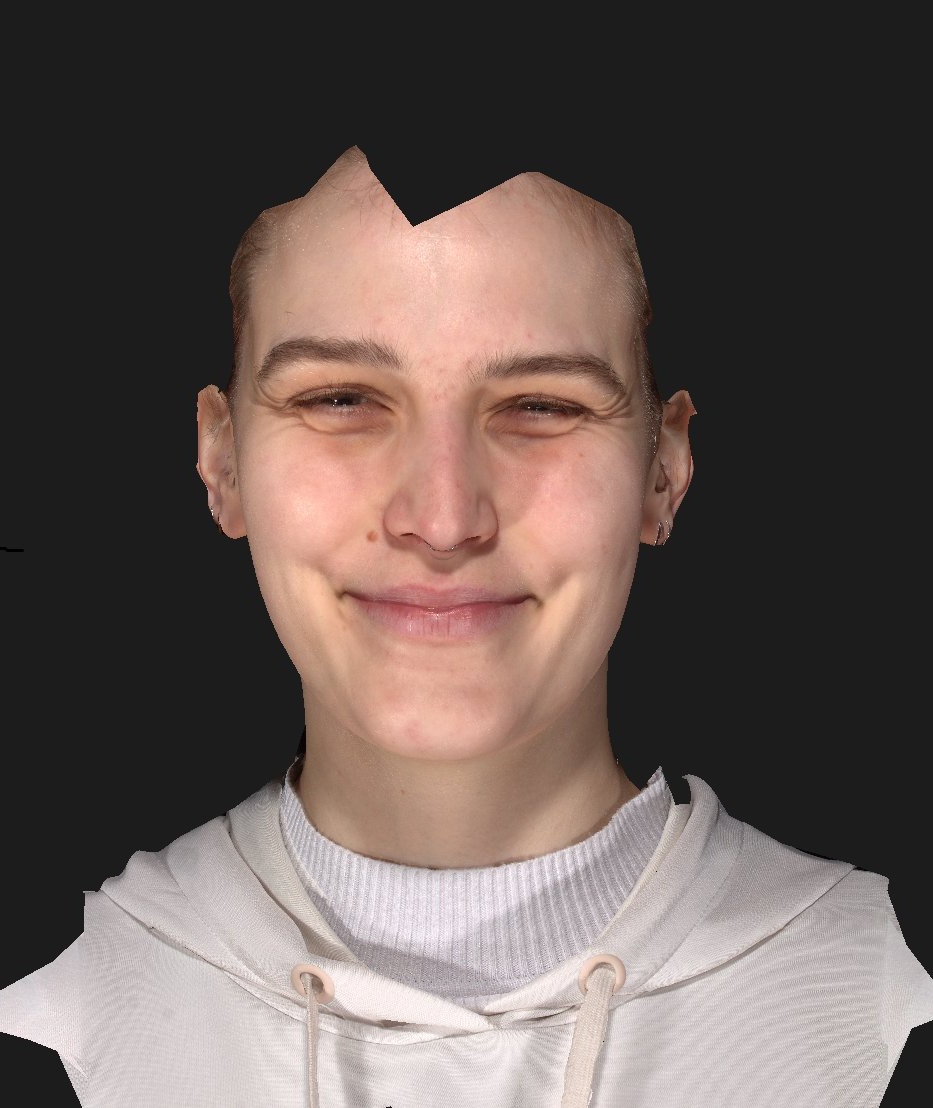

Supplement: ojaf136_Supplementary_Data [file ojaf136_supplementary_data.zip › Supplementary Figure_2A.jpg]

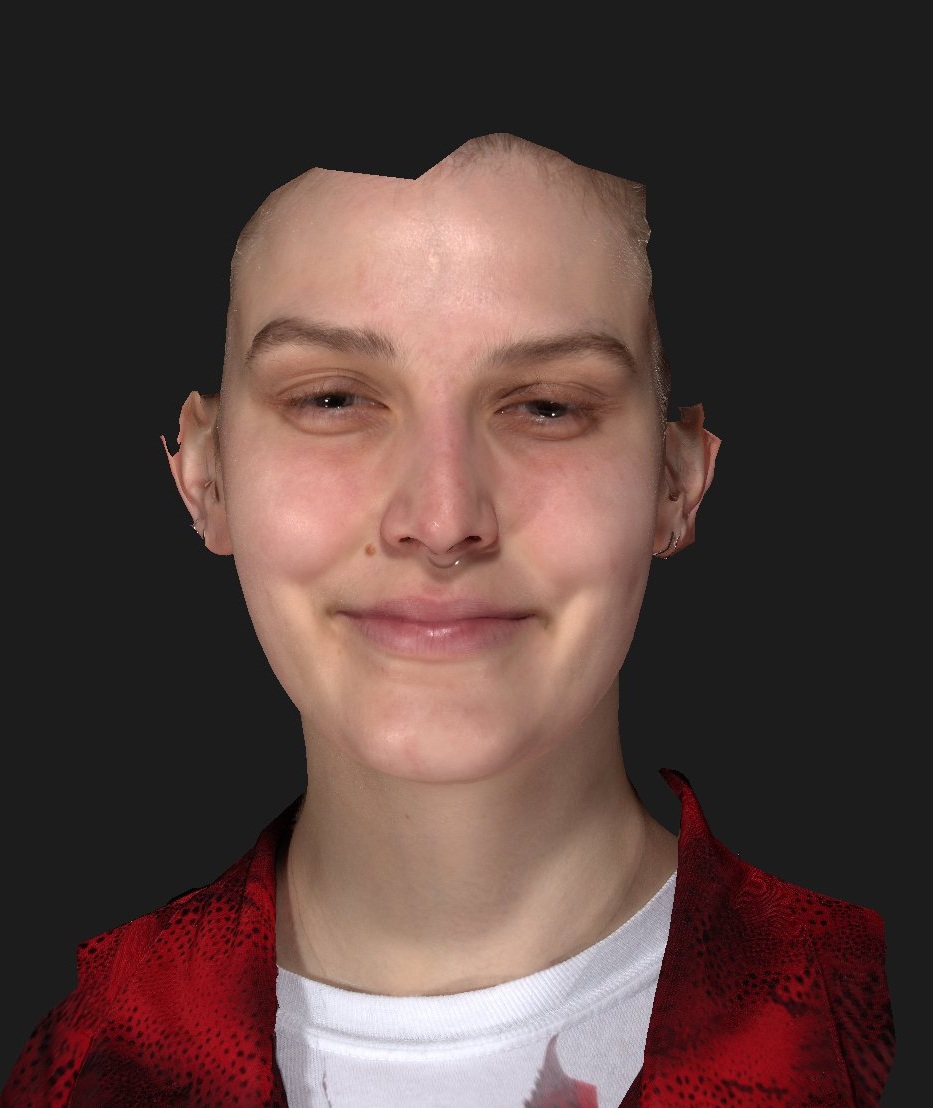

Supplement: ojaf136_Supplementary_Data [file ojaf136_supplementary_data.zip › Supplementary Figure_2B.jpg]

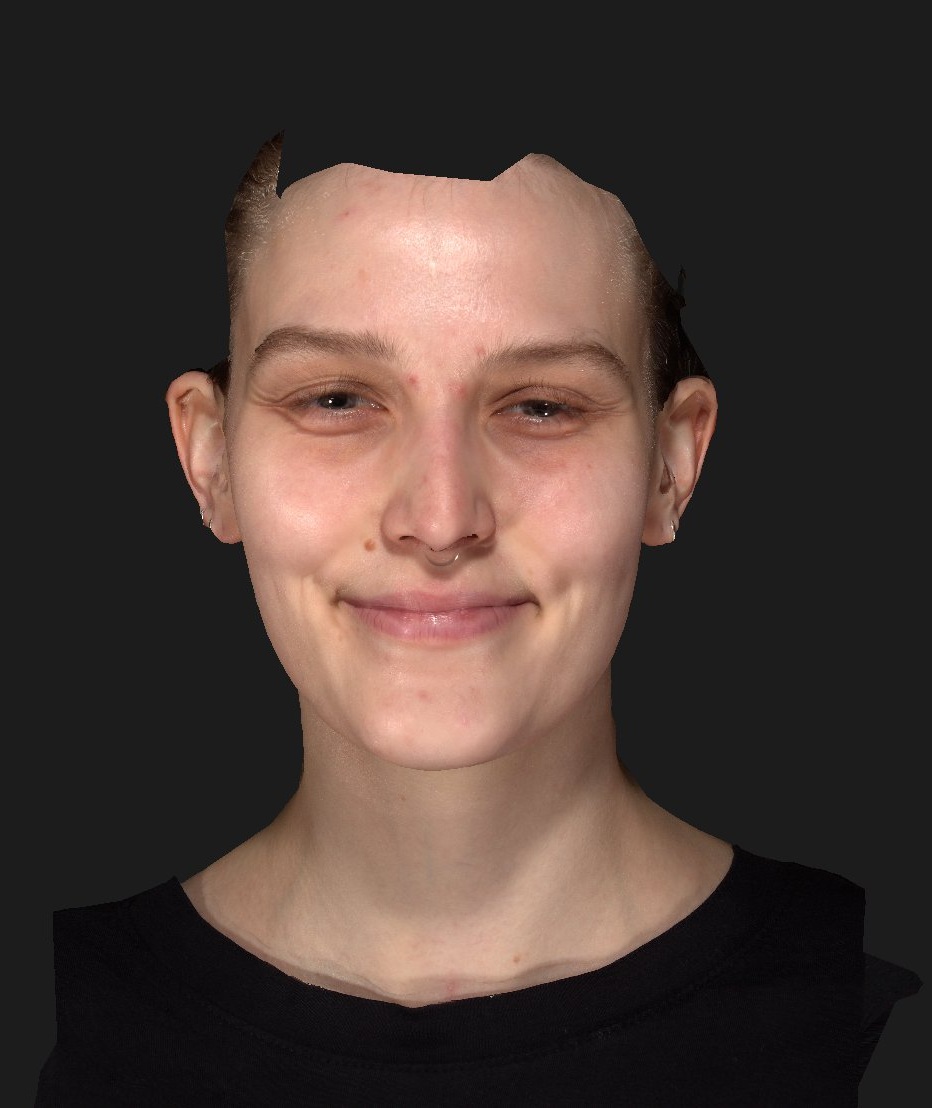

Supplement: ojaf136_Supplementary_Data [file ojaf136_supplementary_data.zip › Supplementary Figure_2C.jpg]
